# Generation of an Enhancer-Trapping Vector for Insertional Mutagenesis in Zebrafish

**DOI:** 10.1371/journal.pone.0139612

**Published:** 2015-10-05

**Authors:** Chunyan Liu, Guili Song, Lin Mao, Yong Long, Qing Li, Zongbin Cui

**Affiliations:** 1 The Key Laboratory of Aquatic Biodiversity and Conservation of Chinese Academy of Sciences; Institute of Hydrobiology, Chinese Academy of Sciences, Wuhan 430072, Hubei, China; 2 University of Chinese Academy of Sciences, Beijing 100049, China; University of Maryland, UNITED STATES

## Abstract

Enhancer trapping (ET) is a powerful approach to establish tissue- or cell-specific reporters and identify expression patterns of uncharacterized genes. Although a number of enhancer-trapping vectors have been developed and a large library of fish lines with distinct tissue- or cell-specific expression of reporter genes have been generated, the specificity and efficiency of trapping vectors need to be improved because of the bias interaction of minimal promoters with genomic enhancers. Accordingly, we generated an enhancer-trapping vector pTME that contains a minimal mouse metallothionein gene (mMTI) promoter upstream of EGFP reporter. In the first round of screening, twelve zebrafish lines that carry a single copy of ET cassettes were characterized to have tissue- or cell-specific EGFP expression. One of the highly conserved noncoding elements near an insertion site of trapping cassettes was characterized as an enhancer that can specifically regulate the expression of EGFP in cells of the central nervous system. In addition, the pTME vector contains a mutation-cassette that is able to effectively block the transcription of an endogenous gene in an ET line with ubiquitous EGFP expression. Thus, the pTME vector can be used as an alternative tool for both enhancer trapping and mutagenesis across a target genome.

## Introduction

Enhancer trapping (ET) is an effective approach widely used to characterize enhancers that control spatio-temporal expression patterns of genes in cells. A basic enhancer-trapping vector is composed of a reporter gene under the control of a minimal promoter [1]. Enhancers in the chromosome cannot drive the expression of a reporter gene in the trapping construct by themselves unless they induce the activity of a minimal promoter [2]. Since the activity of enhancers in a genome is able to regulate the transcription of endogenous genes, the distribution of a reporter in ET cassette can mimic the expression pattern of an endogenous gene and thus makes the ET approach effective to monitor gene activity throughout the genome [3].

ET technology has been successfully used for analysis of gene expression and functions in Drosophila at early stages and the continuous improvement of ET elements allows researchers to obtain a large variety of lines for further research in Drosophila and enable an extensive practice of this method in vertebrate animals, including zebrafish [2]. Zebrafish as a vertebrate model have considerable advantages in ET investigations mainly because fluorescent-protein reporter genes can be used to easily monitor the timing and sites of gene expression [4, 5]. As the development of retrovirus- and transposon-based technologies for gene transfer, the efficiency of reporters integrating to the genome has been definitely improved. Ellingsen and colleagues generated a pseudotyped Moloney murine leukemia virus (MLV)-based trapping construct that contained a basal *gata2* promoter and the YFP reporter gene and obtained 95 transgenic lines with different expression patterns [6]. However, the broad application of this method is impeded because high-titer viruses are hardly created in zebrafish. Alternatively, transposon systems become more popular tools for enhancer detection in zebrafish. A Sleeping Beauty (SB) transposable system containing a s1EF1α promoter and the GFP gene was generated and 9 zebrafish lines with distinct tissue-specific expression patterns were obtained [7]. *Tol2* transposable system, developed by Kawakami and colleagues, is the most popular transposon system used in zebrafish because it has a high transgenic efficiency and it has a propensity to insert into protein-encoding genes [8, 9]. A number of *Tol2*-based ET constructs have been designed and successfully used for generating numerous transgenic lines with tissue- or organ-specific expression of fluorescent reporters [10–16]. In addition to traditional ET vectors that contain a basal promoter and a reporter gene [12, 16], a binary Gal4-UAS system has been successfully used for ET construction and monitoring cell- or tissue-specific expression of reporter genes in Drosophila and zebrafish [2, 10, 11, 13, 15, 17–19].

Despite the success of earlier vectors, there is room to improve ET trapping efficiency, reduce the basal activity of promoters, and add an activity of insertional mutation to ET vectors. Selecting an applicable minimal promoter is essential for the success of constructing an ET cassette. Some of minimal promoters from *ef1a*, *gata2*, *keratin8* (*krt8*), *keratin4* (*krt4*), *hsp70*, *c-fos*, *E1b*, *thymidine kinase* (*TK*), and *carp β-actin* promoters have been applied to ET studies in zebrafish, but they demonstrate distinctive efficiencies in response to genomic enhancers and have biases for specific tissue or cell types [3]. For instance, The *E1b* promoter in an ET construct was found to selectively drive the expression of reporter genes in the cranial ganglia of zebrafish [15]. Moreover, nonspecific background expression of reporter genes induced by basal promoters remains a problem for the broad usage of ET technologies[12]. Although phenotypic mutants from *Tol2* transposon-based ET have been reported [12], in most cases, traditional ET cassette lands in non-coding regions and introns that are generally not suitable for functional characterization of endogenous genes.

In the present study, we constructed a *miniTol2* transposon-based [9, 20] ET vector that contained a mouse metallothionein gene (mMTI) basal promoter [21] and an EGFP reporter In addition, a splicing acceptor (SA) and a partial exon were introduced into the ET cassette for mutagenesis in case of the insertion occurs in an intron of endogenous genes. In the first round of ET screening, twelve ET fish lines were generated and three of them demonstrated an exclusive EGFP expression in the pancreas and kidney. Furthermore, a number of potential enhancer sequences were identified near an insertion site of the ET line pTME12 and one of these sequences exhibited an enhancer activity.

## Materials and Methods

### Ethics statement

The animal protocol used in this study was proved by the Animal Care and Use Committee of Hubei Province in China and the Institutional Animal Care and Use Committee of Institute of Hydrobiology (Approval ID: Keshuizhuan 0829). All sections of this report adhere to the ARRIVE Guidelines for reporting animal research [22]. A completed ARRIVE guidelines checklist is included in S1 Checklist.

### Zebrafish husbandry

The AB strain of zebrafish (*Danio rerio*) was reared in a recirculating water system according to standard protocols [23]. The fish were maintained at 28°C(27°C±2) on a 14 hr:10 hr light:dark cycle (lights on 8 am) in a licensed aquarium facility (ESEN, China). Fish were fed three times a day: twice with brine shrimps (morning and late afternoon) and once with flake food at mid-day. During housing, fish were monitored once daily for health status. No adverse events were observed. Naturally fertilized zebrafish embryos were incubated at 28°C and staged by hour post fertilization (hpf) or day post fertilization (dpf) as described in the Zebrafish Book [23].

### Plasmids

Our ET vector pTME was designed to effectively detect chromosomal enhancers and disrupt the transcriptional expression of endogenous genes under certain circumstances. These elements were sequentially subcloned into the *Tol2* transposon by substitution of the protein trap and 3’ exon trap cassettes in pGBT-RP2.1 [24]. The mMTI minimal promoter was amplified from mouse genome using primers 5’-CAACTCGAGACTCGTCCAACGACTATAAAG-3’ and 5’-GGCCATGGGGTGAAGCTGGAGCTACG-3’ and then inserted at the *Xho*I/*Nco*I site upstream of the EGFP gene. The SA and an exon sequence containing stop codes (TGAATTAGTGA) for three different read frames were obtained from pT2/Gene-Trap vector [25] using primers 5’-AGACTGCAGATTGCAGCACGAAA-3’ and 5’-CGTACGTCACTAATTCAT CATTCACATAC-3’ and ligated at the *Pst*I/*BsiW*I site upstream of mMTI basal promoter.

The pTME-Z48 vector was designed to test the sensitivity of mMTI promoter to genomic enhancers. Z48 enhancer [26] was amplified from a vector (a kind gift from Dr J. Bessa) using primers 5’-TAACGCGTATTAATCCCCCTGCTTCAGC-3’ and 5’-TACATATGGCTAGCGCTCTCGCAGTTG-3’. PCR products were digested with *Mlu*I/*Nde*I and inserted at the locus upstream of the R-ITR of *Tol2* transposon. Four vectors including pTMEt-CNE1, pTMEt-CNE2, pTMEt-CNE3 and pTMEt-CNE4 were constructed to test potential enhancer activity of four conserved noncoding elements (CNEs). CNE1, CNE2, CNE3 and CNE4 were generated from zebrafish genome using primer pairs: CNE1, 5’-CAATCTAGAGCCCACAGATCTGCACTTG-3’ and 5’-GGCTCGAGATTATACCCAAGCAACAACAGG-3’; CNE2, 5’-CAATCTAGACTGCGTCAAACTATAGAAAACTGG-3’ and 5’-GGCTCGAGA ATCATTTATATCTTCCAAACCAGTAAG-3’; CNE3, 5’-CAATCTAGAATCCCA ATCCCAGGCTTTTC-3’ and 5’-GGCTCGAGTCATCTGTGGTCATAGCAG C-3’; CNE4, 5’-CAATCTAGAGTGAGAGGCGACAGCGTGAG-3’ and 5’-GGCTCG AGGGTACAGTGTCTCTCCGAGGGCAG-3’. Next, PCR products for four CNEs were digested with *Xba*I/*Xho*I and inserted upstream of mMTI promoter in the pTMEt vector.

### Microinjection and selection of EGFP-expressing F1 fish

To test the activity and sensitivity of promoters or CNEs, 200 pg of plasmids were injected into each zebrafish embryo at one-cell stage. In each batch of experiments, four pairs of parental fish were used to naturally reproduce fertilized eggs and these eggs were evenly allocated to the control and experimental groups for microinjection. There were approximately 100 eggs in each group. Three batches of independent experiments were conducted to monitor the EGFP expression.

In the ET assays, 50 pg of pTME vectors were co-injected with 100 pg of capped *Tol2* transposase mRNA into each fertilized egg at one-cell stage. Injected embryos with EGFP expression during embryogenesis were reared to adults and individually outcrossed with wide-type fish. All of F1 embryos were collected and screened for EGFP expression from 1 to 7 dpf under a fluorescent microscope Zeiss M205.

### Genome walking and PCR assays

EGFP-expressing F1 adults were individually outcrossed with wide-type fish to obtain F2 embryos and genomic DNAs were extracted from F2 embryos. Genome walking assays were performed according to the Genome Walking Kit protocol from TaKaRa with some modifications to obtain chromosomal DNA sequences flanking the integration sites of *Tol2* transposons. Degenerate primers AP1-4 in the kit were substituted with AD5, AD6 and AD7 in our experiments. *Tol2* R-ITR-specific gwPCR primers were R1, R2 and R3 and *Tol2* L-ITR-specific gwPCR primers were L1, L2 and L3. Primary PCR was performed with primers pairs R1/AD5, R1/AD6, R1/AD7, L1/AD5, L1/AD6 or L1/AD7 under the following conditions: 1 cycle at 94°C for 1 min and 98°C for 1 min; 5 cycles at 94°C for 30 s, 63°C for 1 min and 72°C for 2 min; 1 cycles at 94°C for 30 s, 25°C for 3 min and 72°C for 2min; 15 cycles at 94°C for 30 s, 63°C for 1 min, 72°C for 2 min, 94°C for 30 s, 63°C for 1 min, 72°C for 2 min, 94°C for 30 s, 44°C for 1 min and 72°C for 2 min; 1 cycle at 72°C for 10 min. The first round PCR product (0.5 μL) was used as a template for second round PCR with primer pairs R2/AD5, R2/AD6, R2/AD7, L2/AD5, L2/AD6 or L2/AD7 under the following conditions: 15 cycles at 94°C for 30 s, 63°C for 1 min, 72°C for 2 min; 94°C for 30 s, 63°C for 1 min, 72°C for 2 min; 94°C for 30 s, 44°C for 1 min and 72°C for 2 min; 1 cycle at 72°C for 10 min. Then, 0.5 μl of the second round PCR product was used as a template for the last round PCR with primer pairs R3/AD5, R3/AD6, R3/AD7, L3/AD5, L3/AD6 or L3/AD7 under the same PCR conditions as the second round. The products of the second and third rounds of PCR amplification were loaded on an 1.5% agarose gel. Specific DNA bands in the third round PCR products that were 100-bp smaller than the corresponding products from the second round, which were isolated and cloned into the pZero2/TA vector for sequencing.

PCR assays were conducted to confirm the location of transposon integration in the genome under following conditions: 95°C for 5 min; 34 cycles at 95°C for 30 s, 58°C for 30 s and 72°C for 30 s; 72°C for 10 min. RT-PCR assays were conducted to detect the formation of fusion transcripts as previous described [25]. Total RNAs from wide-type, F1 heterozygous and F2 homozygous embryos were isolated and used for cDNA synthesis according to First Strand cDNA Synthesis Kit from Fermentas. The endogenous transcripts of *skor2* and fusion transcripts were detected using primer pairs E1F/E2R, E1F/ER, E1F2/EGR or E1F2/BR under the following programs: 95°C for 5 min; 34 cycles at 95°C for 30 s, 58°C for 30 s and 72°C for 2 min; 72°C for 10 min. The PCR products were subjected to 1% agarose gel electrophoresis and sequenced.

Quantitative real-time PCR (qPCR) assays were performed to determine the relative levels of EGFP and *skor2* expression as previously described [25, 27]. To test the promoter activity, zebrafish embryos at one-cell stage were microinjected with plasmid pTRE/G2-mMTI, pTRE/G2-SV40, pTRE/G2-EF1α or pTRE/G2-CMV and the injected embryos at 24 and 48 hpf were then pooled for RNA extraction and subsequent qPCR analysis of EGFP expression. To test the sensitivity of mMTI promoter, embryos at one-cell stage were microinjected with plasmid pTME or pTME-Z48 and the injected embryos at 20 hpf were then pooled for RNA extraction and subsequent qPCR analysis of EGFP expression. Primer pairs used in these experiments were *egfp*-qF/*egfp*-qR and *β-actin*-F/*β-actin*-R. To detect the *skor2* expression, total RNAs were extracted from wide-type, heterozygous and homozygous F2 embryos at 48 hpf and converted to cDNA. Then, qPCR was conducted using primer pairs E1F2/E2R and *β-actin*-F/*β-actin*-R. All of qPCR experiments were conducted with RNA extracts from three independent batches of embryos and each reaction was run in triplicate. The expression of *β-actin* was used as the reference to calculate the relative expression of EGFP or *skor2* using the 2^–(ΔΔ Ct)^ method [28].

Primers used for these PCR assays are listed as follows:

AD5: 5’-STAGNATSGNGTNCAA-3’;
AD6: 5’-WGCANGAWGNAGNATG-3’;
AD7: 5’-NTCGTSGNATSTWGAA-3’;
R1:5’-GTACAATTTTAATGGAGTACTTTTTTACTTTTACTCAAG-3’;
R2: 5’- CCAGATACTTTTACTTTTAATTGAGTAAAATTTTCC-3’;
R3: 5’-CACTTGAGTAAAATTTTTGAGTACTTTTTACACC-3’;
L1: 5’-GGTTTGGTAATAGCAAGGGAAAATAGAATG-3’;
L2: 5’-GATTTTTAATTGTACTCAAGTAAAGTAAAAATCCCC-3’;
L3: 5’-CAGTAATCAAGTAAAATTACTCAAGTACTTTACACC-3’;
ER: 5’-CACATACCGGCTACGTTGCTAAC-3’;
RF:5’-ATGTGTCTTCATCTGAAAAGCGTTC-3’;
LR: 5’-GTACATCCTTGCTCAGAATCTCCCTC-3’;
*egfp*-qF: 5’-GCAGAAGAACGGCATCAAGG-3’;
*egfp*-qR: 5’-CGGACTGGGTGCTCAGGTAG-3’;
*β*-*actin*-F: 5’-CGAGCAGGAGATGGGAACC-3’;
*β*-*actin*-R: 5’-CAACGGAAACGCTCATTGC-3’;
E1F: 5’-GGACTAGGAGCAGGAAGAC-3’;
E1F2: 5'–CTTCCTGACAGCCAAACAACAG-3';
E2R: 5’-GGCGGCTGTTTTGTTTTTGG-3’;
I1F: 5’-ACAGACAGATCGAGAAGAGCGAC-3’;
EGR: 5’-ACTTGTGGCCGTTTACGT-3’;
BR: 5’-ACAGATGGCTGGCAACTAGAAG-3’.

### Computational analyses

The chromosomal flanking DNA sequences around the insertion sites were mapped on the zebrafish genome sequence (Zv9) in the ENSEMBL by BLASTN. Genomic sequences spanning the region between *rhcga* and *kif7* gene from Fugu, Medaka and zebrafish were downloaded from the Ensemble browser (www.ensembl.org), and multiple alignments were carried out by mVISTA analysis (http://genome.lbl.gov/vista/mvista/submit.shtml).

### Whole-mount RNA *in situ* hybridization

Antisense RNA probes for *mecom*, *hs3st3b1b*, *egfp*, *tcf7l2* and *skor2* were synthesized by using digoxigenin (Roche) as a label. Whole-mount RNA *in situ* hybridization (WISH) was performed as previously described [29]. Images were captured using the SteReo Lumar V12 from Carl Zeiss.

### Southern blot hybridization

Southern blotting of genomic DNA from F2 embryos was performed following our previous protocol [25]. A total of 20 μg genomic DNA was digested with *Bgl*II or *Pst*I (a unique restriction site in the pTME vector). The probes were amplified from EGFP coding sequence with primers TZ-EGFP-f 5’-ATGGTGAGCAAGGGCGAGGAGC-3’ and TZ-EGFP-r 5’-ACGCTGCCGTCCTCGATGTTGT-3’.

### Statistical Analysis

Microsoft Excel was used to derive standard deviation and perform student’s t-tests.

## Results

### Construction of a novel enhancer trapping vector

**Fig 1:**
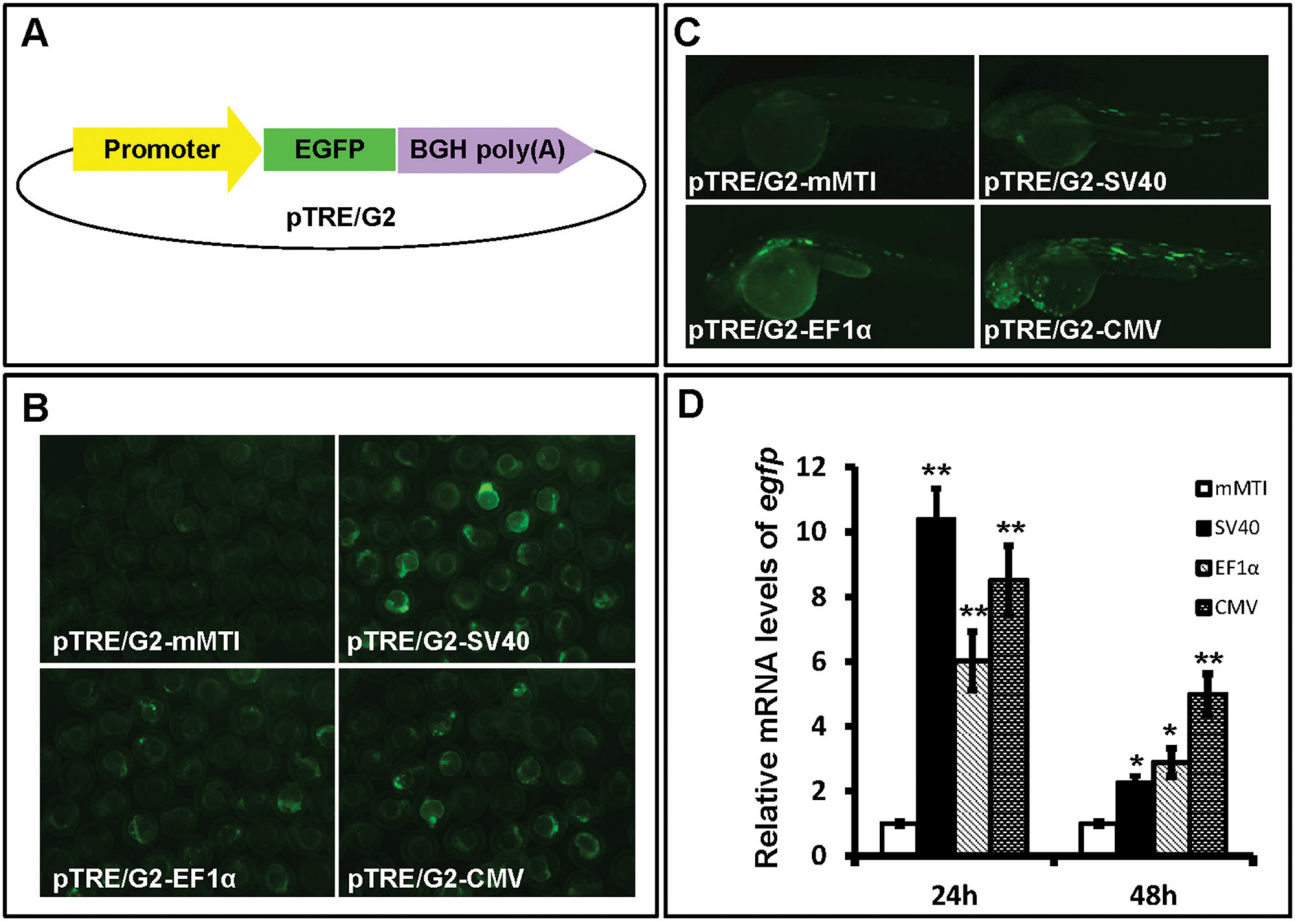
Expression of minimal promoters from mMTI, SV40, EF1α and CMV. **(A)** The schematic drawing of pTRE/G2 vector used for testing promoter activity. **(B and C)** Representative images of embryos injected with pTRE/G2 vectors containing mMTI, SV40, EF1α or CMV minimal promoters under a fluorescence microscope at 24 and 48 hpf. **(D)** Expression of EGFP was detected by qRT-PCR in injected embryos at 24 and 48 hpf. Data were calculated from three independent experiments and given as means ± standard deviation. * and ** indicate p<0.05 and p<0.01 *versus* the group injected with pTRE/G2-mMTI embryos.

In this paper, we generated a novel ET vector pTME that was meditated by the *miniTol2* transposon system. To obtain an appropriate minimal promoter with a lower basal activity, we first tested the activities of four minimal promoters from mMTI, CMV, SV40 and truncated EF1α promoters. These promoters were subcloned into a pTRE/G2 EGFP reporter vector, and then zebrafish embryos at one-cell stage were injected with these modified vectors (Fig 1A). Embryos were observed for EGFP expression at 24- and 48-hours post-fertilization (hpf). We found that embryos injected with PTRE/G2 vector containing mMTI minimal promoter exhibited the weakest EGFP expression in comparison with those injected with vectors containing other minimal promoters (Fig 1B and 1C). Levels of EGFP were detected by quantitative RT-PCR and the results verified that the mMTI exhibited the lowest expression activity than other three minimal promoters (Fig 1D). Assuming that expression is proportional to the transcriptional power of the selected sequences, we conclude that the mMTI minimal element was appropriate for our needs and accordingly used in our ET vector pTME.

**Fig 2:**
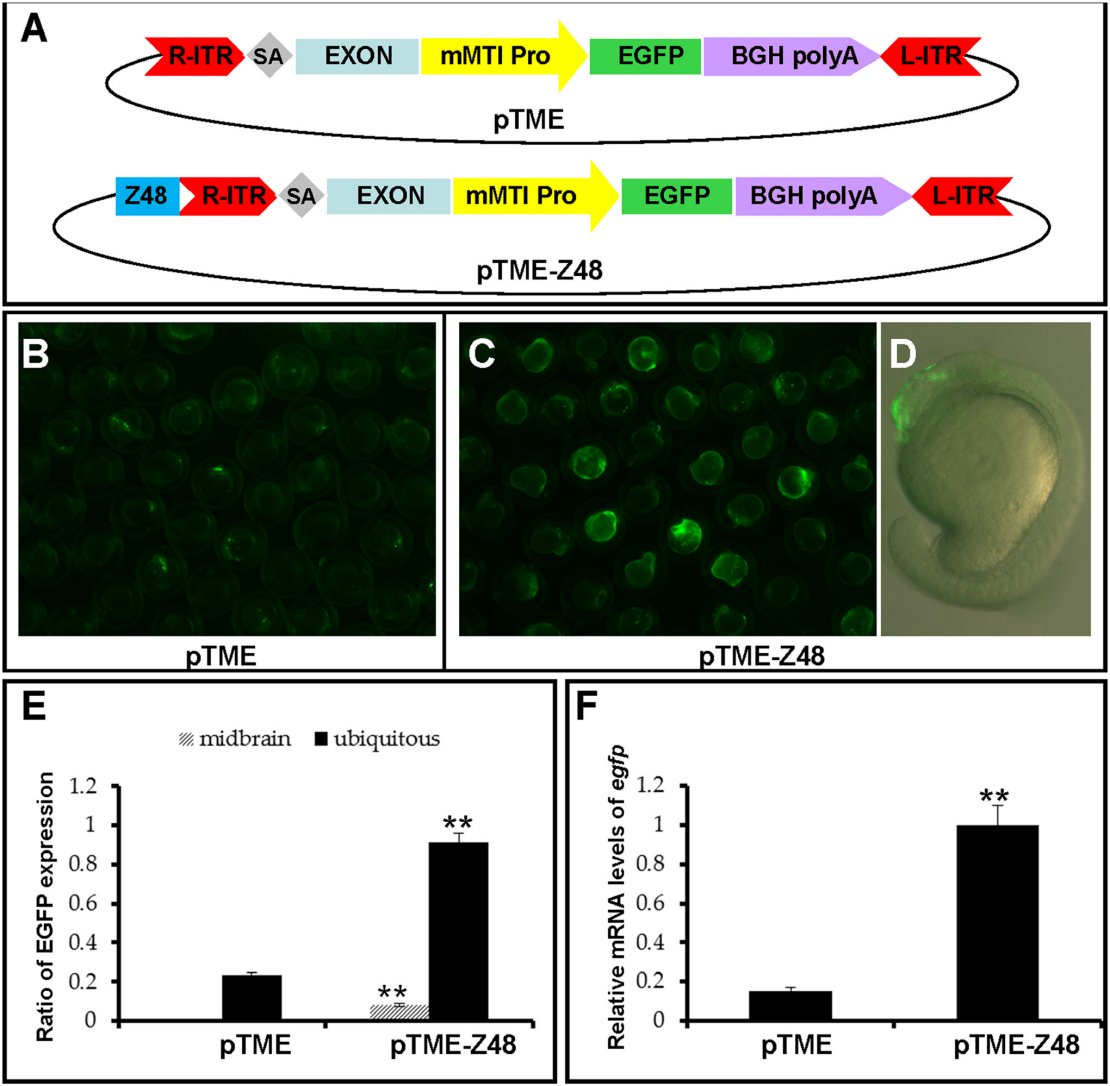
Artificial assays of ET activity of mMTI promoter. **(A)** ET vector pTME and an artificial ET vector pTME-Z48 in *miniTol2* transposons. ITR-R and ITR-L, right and left inverted terminal repeats of *miniTol2* transposon; SA, splice acceptor from carp *β-actin* gene; Exon, exon2 from the carp *β-actin* gene; mMTI Pro., mMTI minimal promoter; BGH polyA, bovine growth hormone polyadenylation sequence. The Z48 enhancer was subcloned into the pTME vector upstream of ITR-R. **(B and C)** Zebrafish embryos were injected with pTME or pTME-Z48 at the one-cell stage and images were captured under a fluorescent microscope Zeiss M205 at 20 hpf. **(D)** The midbrain-specific expression of EGFP in one representative embryo from (C) injected with pTME-Z48 is shown in a merged fashion. **(E)** The average percentage of embryos with ubiquitous or midbrain specific GFP fluorescence in three independent experiments. Total numbers of embryos injected with pTME or pTME-Z48 were counted at 20 hpf and scored for ubiquitous EGFP expression and specific EGFP expression in the midbrain. **(F)** qRT-PCR analysis of relative EGFP mRNA levels in injected embryos. Data are given as means ± standard deviation (n = 3). ** indicates *P* < 0.01 *versus* the pTME-injected embryos.

To examine whether the mMTI minimal promoter can be activated by nearby enhancers, an ET event was artificially mimicked by addition of a midbrain-specific enhancer Z48 proximal to the right arm of *miniTol2* inverted terminal repeats (R-ITR) in the pTME vector (Fig 2A). As shown in Fig 2E, 8.3% (29/354) of pTME-Z48-injected embryos exhibited midbrain-specific EGFP expression (Fig 2D). These data showed that expression from the mMTI minimal promoter could be regulated by an adjacent enhancer such as Z48 that mimicked faithful tissue-specific EGFP expression in developing embryos of zebrafish. However, an unexpected increase of ubiquitous EGFP expression was observed in pTME-Z48-injected embryos. 91% (293/322) of pTME-Z48-injected embryos displayed ubiquitous EGFP expression, while the ratio in pTME-injected embryos is 24% (84/354) (Fig 2B, 2C and 2E) and the EGFP expression level in pTME-Z48-injected embryos was about ten times higher than that in pTME-injected embryos at 20 hpf (Fig 2F). Such an increase of ubiquitous EGFP expression in pTME-Z48-injected embryos may be caused by the position of Z48 enhancer since the distribution of enhancers outside the transposon systems in a transgenic vector could lead to more mosaic expression [7, 30].

### Enhancer screening with pTME

Zebrafish embryos at one-cell stage were co-injected with the pTME vector and capped *Tol2* transposase mRNAs. Most of the injected embryos can survive to 3 dpf and about one third of the injected embryos that showed EGFP expression were raised for further analysis. During the first round of ET screening, 298 embryos survived to adulthood and these founder adults were separately outcrossed with wide type (WT) fish for germline-transmission analysis. 235 founders were fertile and 75 of them produced F1 offspring with EGFP expression, which gives an transgenic rate of about 32% (Table 1). Next, F1 offspring from 10 founders that exhibited distinct patterns of tissue-specific EGFP expression were used to establish specific ET lines. Twelve ET lines (pTME1-12) with tissue-specific EGFP expression were separately outcrossed with WT fish to generate F2 offspring for stable transgenic analysis.

**Table 1:** Overview of the first round ET screening.

Items	Data
F0 fish outcrossed with WT	235
F0 fish with EGFP expression in F1 offspring	75
Transmission rate of F0 fish	32%
Potential ET lines (F1)	12
ET ratio of F0 fish	5%
ET ratio of transgenic F1 fish	16%
Insertions occurred within transcribed regions	4

**Fig 3:**
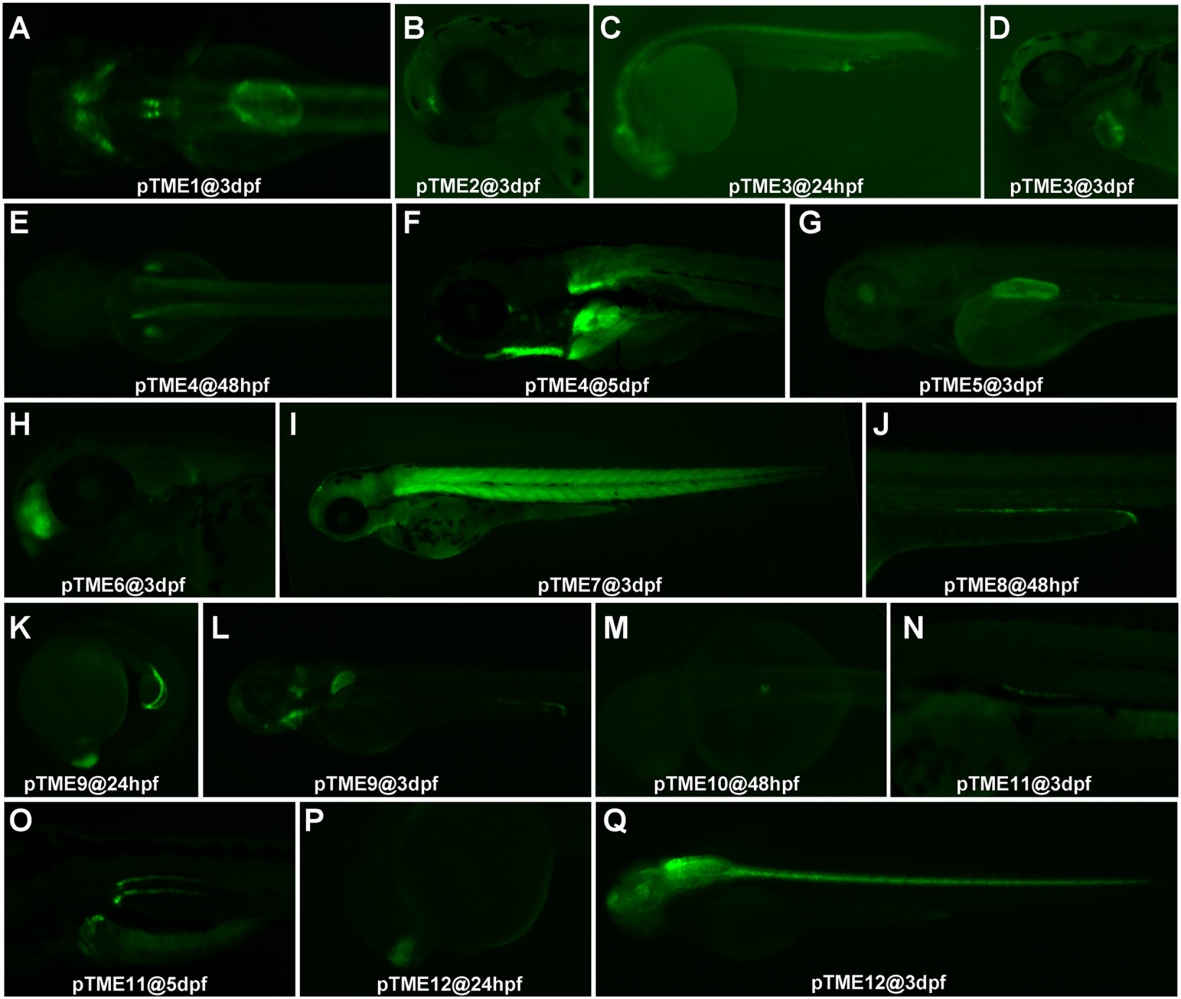
ET lines with different patterns of EGFP expression. **(A)** Dorsal view of pTME1 embryo at 3 dpf. **(B)** Lateral view of pTME2 embryo at 3 dpf. **(C and D)** Lateral view of pTME3 embryos at 24 hpf (C) and 3 dpf (D). **(E and F)** Dorsal view of pTME4 embryos at 48 hpf (E) and lateral view at 5 dpf (F). **(G)** Lateral view of pTME5 embryo at 3 dpf. **(H)** Lateral view of pTME6 embryo at 3 dpf. (**I)** Lateral view of pTME7 embryo at 3dpf. **(J)** Lateral view of pTME8 embryo at 48 hpf. **(K and L)** Lateral view of pTME9 embryos at 24 hpf (K) and 3 dpf (L). **(M)** Dorsal view of pTME10 embryo at 48 hpf. **(N and O)** Lateral view of pTME11 embryos at 3 dpf (N) and 5 dpf (O). **(P and Q)** Lateral view of pTME12 embryos at 24 hpf (P) and 3 dpf (Q).

Line pTME1 represented a symmetric pattern of EGFP expression in the midbrain and rhombomeres at 48 hpf and then in the swim bladder and intestine at 3 dpf (Fig 3A). In pTME2, a single patch of EGFP expression was found in the forebrain ventricle zone at 3 dpf (Fig 3B). In pTME3, a weak EGFP signal appeared in the brain, spinal cord and protodeum at 24 hpf, which gradually declined from 48 hpf (Fig 3C), and then exhibited a heart-specific EGFP expression from 48 hpf to 7 dpf (Fig 3D). In pTME4, EGFP expression was bilaterally distributed in the pectoral fins and myotomes at 48 hpf (Fig 3E) and relatively strong EGFP signals appeared in the pectoral fins as well as the ceratohyal at 5 dpf (Fig 3F). Weak EGFP signals appeared in the swim bladder bud of line pTME5 at 48 hpf and the expression region magnified as the swim bladder expanded through 7 dpf (Fig 3G). In pTME6, strong EGFP expression in the telencephalon and posterior part of otic vesicle was observed from 48 hpf to 7 dpf (Fig 3H). EGFP expression in pTME7 came out in the myotomes at 48 hpf and remained a more pronounced expression in this area and the dorsal diencephalon as well as the branchial arches at 72 hpf (Fig 3I). In pTME8, EGFP was seen in the posterior part of pronephric ducts from 48 hpf to 7 dpf (Fig 3J). EGFP in pTME9 was noticed in the telencephalon and posterior part of pronephric ducts at 24 hpf (Fig 3K) and faded away from the telencephalon at 48 hpf, but remained strong in the hindbrain, cranial ganglia, branchial arches, pectoral fin and posterior part of pronephric duct until 3 dpf (Fig 3L). By 4 dpf, EGFP was diminished in the hindbrain and the pronephric duct, but enhanced in the cranial ganglia, pectoral fin, and the pharyngeal arches. EGFP expression in pTME10 first appeared in the endocrine pancreas at 24 hpf and was restricted to the region until 5 dpf (Fig 3M). A weak EGFP expression was bilaterally observed in the pronephric ducts of pTME11 at 48 hpf and then exhibited a more pronounced expression in the middle of the pronephric ducts at 72 hpf (Fig 3N). A robust EGFP expression in this line was noticed in the anterior area of pronepheric ducts at 5 dpf (Fig 3O). In pTME12, EGFP signals first emerged in the telencephalon at 16 hpf, and expanded to the olfactory placodes and optic stalks at 24 hpf, and remained in these area through 5 dpf (Fig 3P). In addition, we observed weak EGFP expression in the midbrain and hindbrain at 42 hpf, and enhanced expression in the brain and spinal cord at 48 hpf, and EGFP expression in the retina from 72 hpf to adult (Fig 3Q).

Thus, distinct patterns of EGFP expression in ET lines from the enhancer trapping screening indicate that our ET screening approach has the potential of trapping enhancers that control the transcription of multiple endogenous genes in zebrafish.

### Identification of insertion sites in ET lines

To clarify genetic backgrounds corresponding to ET lines with specific EGFP expression pattern, we detected insertion events in twelve ET lines. First, GFP-positive F1 or F2 fish were outcrossed with WT fish to obtain GFP-positive offspring for analyzing copy numbers of ET cassettes by Southern blotting. Next, genomic DNA from fish lines with a single copy of the *Tol2*-transposon was prepared for characterization of insertion sites in the chromosome by PCR-based genome walking. Insertion sites of ET cassettes were determined in eleven ET lines, all except for pTME4, in which the flanking sequence was not successfully mapped to the zebrafish genome (Zv9, Table 2). Data by blasting the Ensemble transcripts database indicate that 40% of insertion events (4/11) occurred within transcribed regions, which is in consistence with those (33% and 39%) from two previous studies using *Tol2*-transposon system [12, 14].

**Table 2:** Insertion sites in twelve of ET lines.

ET Lines	Nearest gene	Chromosome	Location	Orientation
pTME1	ENSDARG00000004415(*tcf7l2*)	12	13kb(upstream)	R
pTME2	ENSDARG00000092826(si:ch211-243g18.3)	10	52kb(downstream)	R
pTME3	ENSDARG00000086645(*hs3st3b1b*)	12	79kb(downstream)	R
pTME4	NA	NA	NA	NA
pTME5	ENSDARG00000009313(*napsa*)	3	Intron 3	R
pTME6	ENSDARG00000039647(*slc6a1b*)	11	3kb(upstream)	F
pTME7	ENSDARG00000093492(si:dkey-246i21.1)	23	Intron 2	R
pTME8	ENSDARG00000040151(*esrrb*)	17	110kb(upstream)	R
pTME9	ENSDARG00000060808*(mecom*)	15	16kb(upstream)	F
pTME10	ENSDARG00000087446*(mmd2a*)	3	Intron 1	R
pTME11	ENSDARG00000018192*(ubr5*)	16	Intron 55	R
pTME12	ENSDARG00000033099*(kif7*)	7	45kb(downstream)	R

Notes: NA indicates the cloned sequence from genome walking PCR was not mapped on the genome (Zv9). F and R represent the insertion occurred in a forward and reverse direction of the nearest gene, respectively.

### EGFP distributions and expression patterns of endogenous genes near the insertion sites in three ET lines

To learn whether EGFP signals in ET lines represent expression patterns of endogenous genes, the identified genetic loci around the ET insertion sites were determined from ZFIN (http://zfin.org).

**Fig 4:**
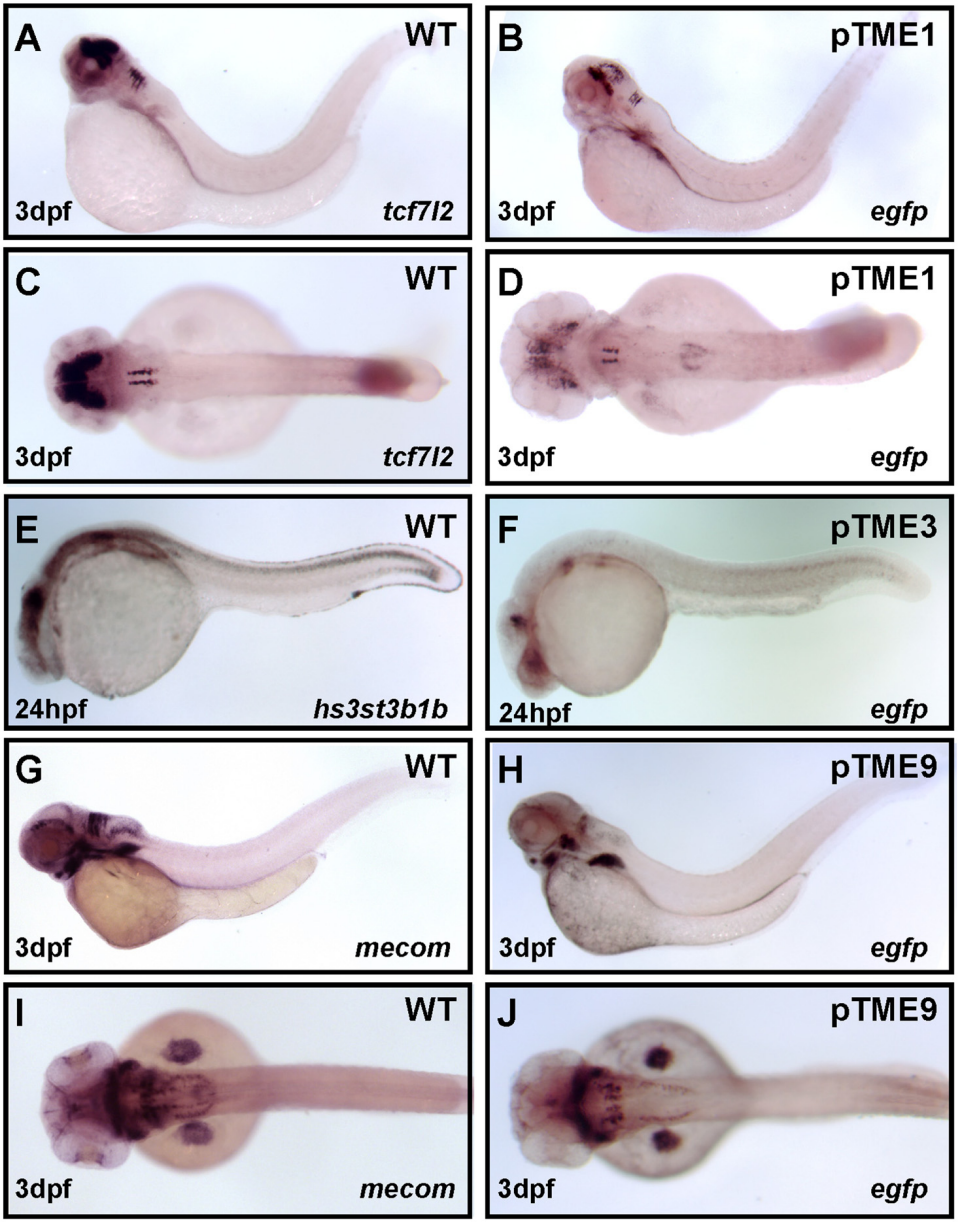
Comparison of EGFP expression patterns in three ET lines with those of corresponding endogenous genes. **(A, B, C and D)** Wide-type (WT) and pTME1 embryos were collected at 3 dpf and WISH assays were performed with antisense RNA probes for *tcf7l2* or *egfp*, respectively. **(E and F)** WT and pTME3 embryos were fixed at 24 hpf for WISH assays. WT embryos were detected with probe *hs3st3b1b* (E) and pTME3 embryos with probe *egfp* (F). **(G, H, I and J)** WISH assays for WT or pTME9 embryos at 3 dpf were conducted with antisense RNA probes for *mecom* or *egfp*. Embryos were viewed in lateral (A, B, E, F, G and H) and dorsal (C, D, I and J).

In pTME1 line, the ET cassette is located at about 13 kb upstream of the protein encoding region of transcription factor 7-like 2 (*tcf7l2*, also known as *tcf4*). Tcf7l2 is a member of T-cell factor Tcf/Lef family whose gene products have binding sites that are commonly found downstream of genes in canonical Wnt signaling pathways [31]. Transcripts of *tcf7l2* first appear in the anterior brain and subsequently are distributed in the dorsal diencephalon, anterior midbrain and rhombomeres of the hindbrain as well as in developing intestine at 3 dpf [32, 33]. EGFP expression patterns in pTME1 (Fig 3A) are similar to those of *tcf7l2* expression in previous studies and WISH was conducted to verify the relativity between this line and *tcf7l2*. Both *egfp* and *tcf7l2* mRNAs were bilateraly distributed in the diencephalon, midbrain and rhombomeres. We also observed obvious expression of *egfp* and *tcf7l2* mRNA in the gut by 3 dpf. However, only *egfp* transcripts were detected in the swim bladder (Fig 4A–4D). In short, EGFP mRNA expression in pTME1 perfectly recapitulates the expression patterns of zebrafish *tcf7l2* gene during embryogenesis.

In pTME3, the ET cassette is about 79kb downstream of the gene encoding heparan sulfate (glucosamine) 3-O-sulfotransferase 3B1b (*hs3st3b1b*). In a previous study, the expression of *hs3st3b1b* (also known as 3-OST-3Z) in the brain, spinal cord, pectoral fin, lateral line primordial, proctodeum, and dorsal fin at 24 hpf was investigated [34]. In line pTME3, weak EGFP expression was also observed in the brain, spinal cord and protodeum at 24hpf, which is similar to the *hs3st3b1b* expression patterns. In order to clearly compare the expression patterns of *hs3st3b1b* with EGFP in pTME3, WISH was performed to detect *hs3st3b1b* expression in WT embryos and EGFP expression in pTME3 embryos at 24 hpf using antisense probes *hs3st3b1b* or *egfp*, respectively. Similar expression patterns of *hs3st3b1b* were shown as previous described (Fig 4E), whereas less *egfp* transcripts were detected in the brain, spinal cord, pectoral fin (Fig 4F). Combined with EGFP fluorescent expression (Fig 3C) and *egfp* mRNA distribution (Fig 4F), we noticed that weak EGFP distribution patterns in pTME3 mimicked the expression patterns of endogenous *hs3st3b1b* gene.

In pTME9, the insertion site of ET cassette was in a non-coding region within 16 kb upstream of the *mecom* transcriptional start site. The *mecom* gene, also known as *prdm3*, or *evi1*, has been reported to express in the posterior pronephric ducts from 20 to 28 somites [35] and in the hindbrain, tagmentum, branchial arches as well as pectoral fin buds from 48 hpf to 72 hpf [36]. The *prdm3* gene also has been shown to express in the pharyngeal arches at 60 hpf and play an critical role in the formation of the craniofacial skeleton in zebrafish [37]. We found that the *mecom* expression patterns as described in these papers are similar to the EGFP expression in pTME9. To confirm whether the EGFP expression in pTME9 mimics the endogenous *mecom* expression, *egfp* and *mecom* RNA probes were separately used to detect *egfp* or *mecom* mRNA expression by WISH in pTME9 or WT larval at 3 dpf. Both *egfp* and *mecom* transcripts were detected in the hindbrain, cranial ganglia, branchial arches and pectoral fin. However, the *mecom* gene is expressed in the tagmentum at 3 dpf but not *egfp* (Fig 4G–4J). Taken together, the *egfp* mRNA distribution in pTME9 largely mimics the expression pattern of the endogenous *mecom* gene.

EGFP expression patterns in two lines, pTME6 and pTME8, are partly similar to the endogenous genes around their insertion sites. The nearest gene *slc6a1b* around the insertion site in pTME6 is normally expressed in the telencephalon as well as retina, midbrain, hindbrain and spinal cord at 72 hpf [38]; the EGFP signals we observed in pTME6 are constrained to the telencephalon at 72 hpf. The endogenous gene *esrrb*, 110 kb downstream of the pTME8 integration site, is expressed in the brain and posterior part of pronephric ducts and the location in the pronephric ducts is the same as EGFP expression in pTME8 fish [38].

Thus, EGFP distributions are similar to expression patterns of genes near insertion sites of the ET cassettes in pTME1, pTME3 and pTME9 lines.

### An enhancer found near the insertion site of the pTEM12 ET line

**Fig 5:**
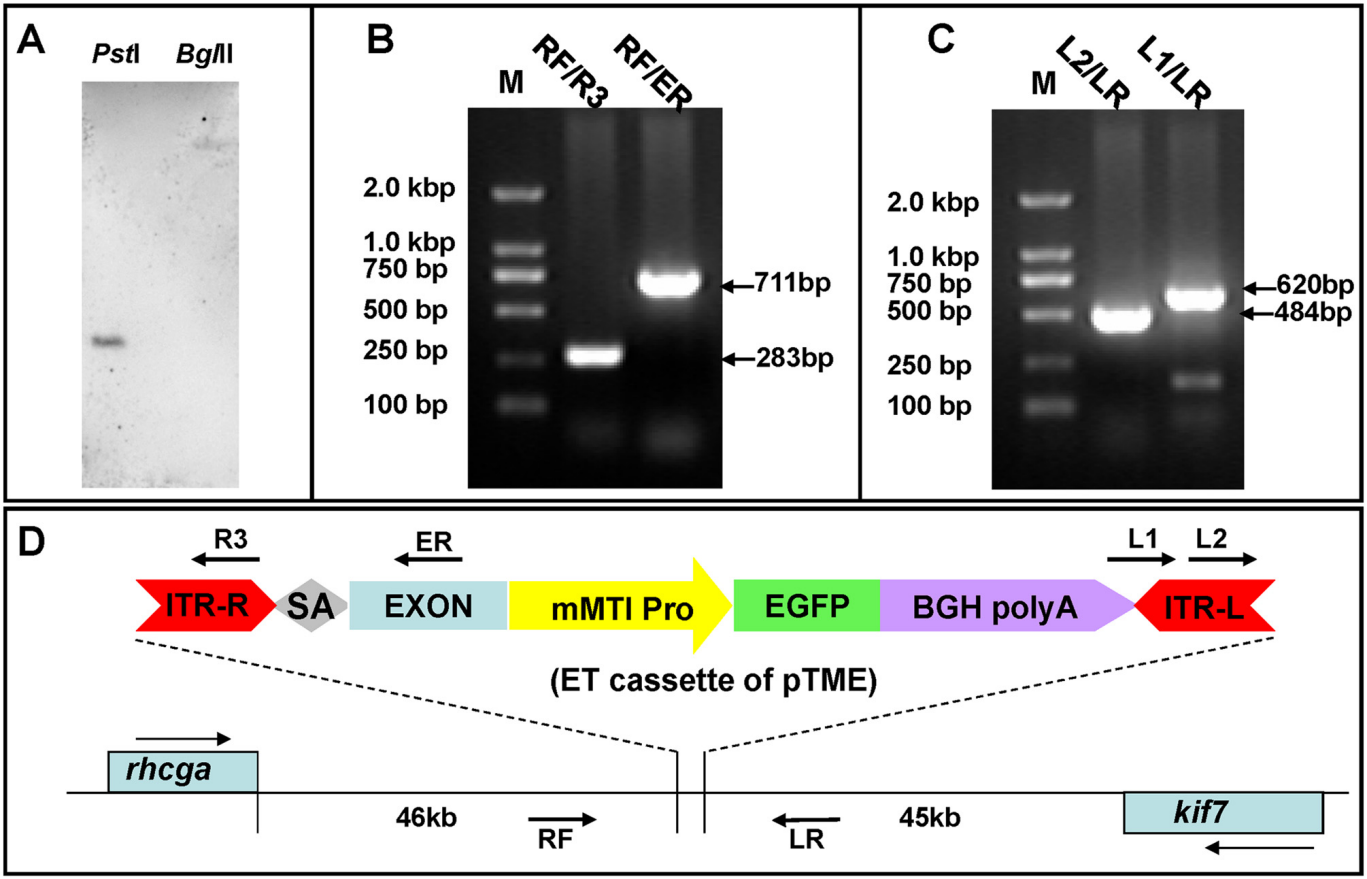
Molecular characterization of genetic background in ET line pTME12. **(A)** Southern blotting for analysis of ET cassette copy number in line pTME12. The genomic DNA was extracted from F2 embryos and digested with *Pst*I and *Bgl*II. **(B and C)** Analysis of an ET cassette in line pTME12. Genomic DNA from F2 embryos was prepared as a template for PCR analysis using primers indicated in (D). **(D)** The sketch of ET cassette location in zebrafish genome. The insertion is located about 46 kb downstream of *rhcga* and about 45 kb downstream of *kif7*. Arrows on top of *rhcga* or under *kif7* indicate transcriptional direction of these genes. The positions of primers used for molecular analysis are showed in the diagram.

In pTME12, a strong EGFP expression was specifically noticed in the central nervous system (CNS) with no background EGFP expression from the minimal promoter elsewhere during embryonic development, a series of experiments were performed to detect potential cis-regulatory elements that function in pTME12. First, we detected the copy numbers of ET-cassette in pTME12. Genomic DNA from F2 offspring was extracted and digested with restriction endonuclease *Pst*I or *Bgl*II, respectively. Southern blotting analysis with EGFP probes revealed a single band of ET-insertion in both enzyme-digested DNA samples (Fig 5A), indicating that a single copy of EGFP-containing ET cassette had inserted in the genome of heterozygotic F2 offspring.

To determine the location of ET-cassette in the genome, total genomic DNA analyzed by genome walking PCR. DNA sequencing indicated that the ET-element had inserted at a non-coding region about 46-kb downstream of *rhcga* in the forward direction and 45-kb downstream of *kif7* in the reverse direction (Fig 5D). To further determine the integrity of inserted ET element, PCR was performed with primers on ET-element and genome near the insertion site. As shown in Fig 5B, a 283-bp DNA band was obtained with primers (RF/R3) and a 711-bp band was amplified with primers (RF/ER). Sequencing results indicated that the left end of ET-element accurately integrated to the genome. In addition, a 484-bp PCR product was amplified with a primer pair (L2/LR) and a 620-bp band was mainly generated with primers (L1/LR) (Fig 5C). Sequence data indicated that the right arm of ET-element landed at the genome as expected. Taken together, the ET cassette indeed inserted in a non-coding region that was 45kb away from the nearest gene *kif7* in pTME12. However, the normal expression patterns of neither *kif7* [39] nor *rhcga* [40] are consistent with EGFP expression patterns we observed in this line.

**Fig 6:**
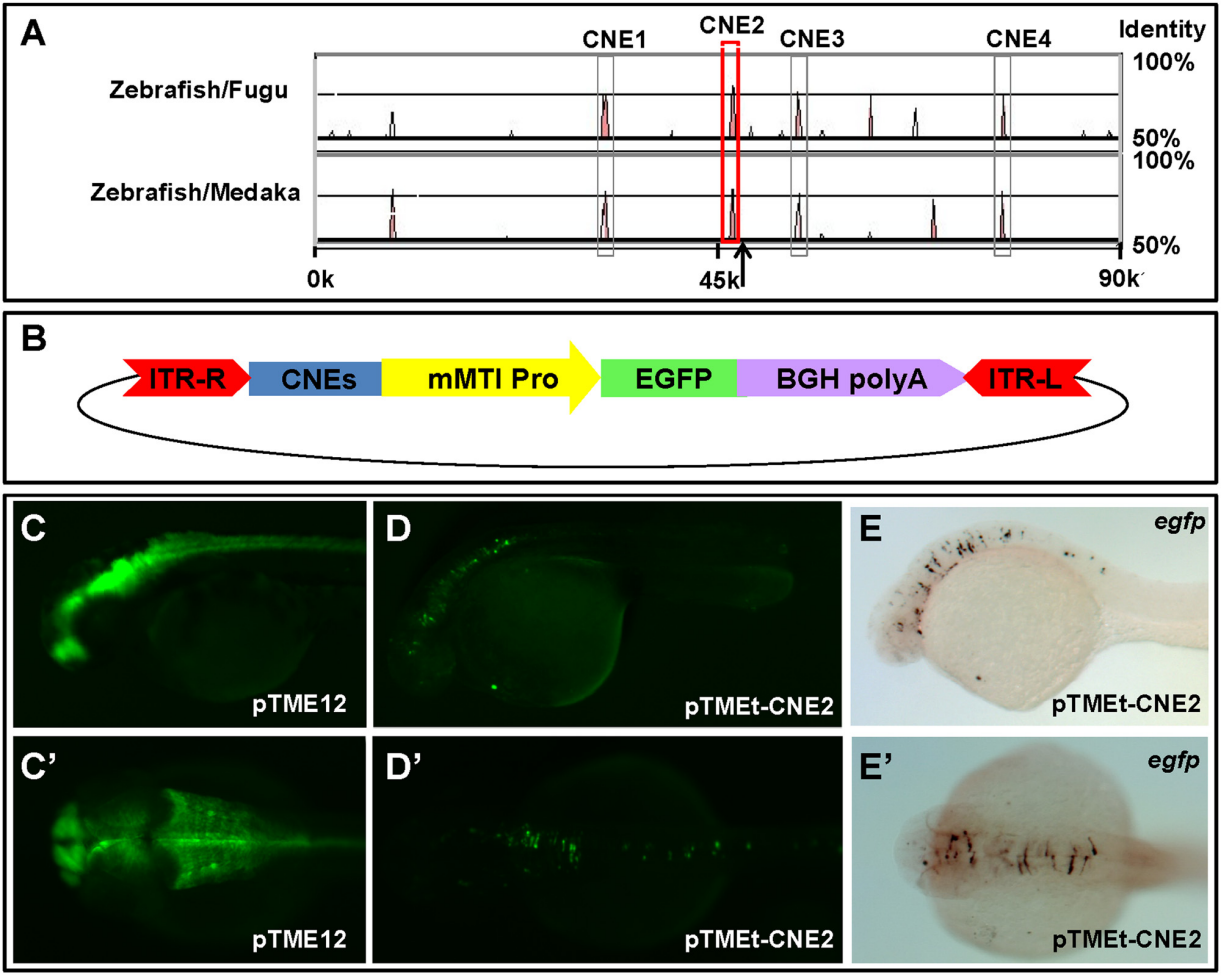
One of conserved noncoding elements (CNEs) near the ET insertion site in pTME12 exhibited an enhancer activity. **(A)** Distribution of CNEs in the region between *rhcga* and *kif7* in zebrafish genome. Genomic sequences between *rhcga* and *kif7* from zebrafish, fugu and medaka were subjected to VISTA browser and four CNEs were predicted in zebrafish genome as indicated (gray and red frame). The location of insertion site is marked with black arrow. **(B)** Schematic representation of pTMEt vector used for testing enhancer activity. Elements used in this vector are the same as pTME except for a substitution of the mutation cassette with one of CNEs. **(C, C’, D, D’, E and E’)** Zebrafish embryos injected with pTMEt-CNE2 exhibited transient and specific EGFP expression in the central nervous system at 48 hpf. EGFP expression in embryos from the ET line pTME12 was viewed in lateral (C) and in dorsal (C’) at 48 hpf. Transient EGFP expression in WT embryos injected with pTMEt-CNE2 and embryos were viewed from lateral (D) and dorsal (D’). Embryos injected with pTMEt-CNE2 were prepared for WISH with *egfp* RNA probes and lateral (E) and dorsal (E’) views of a representative embryo indicate the specific GFP expression in the brain and spinal cord.

A large number of highly conserved noncoding elements (CNEs) in the vertebrate genome have been found to act as tissue-specific enhancers for the regulation of embryonic development [41–45]. To detect enhancer elements near the integration site, a comparative analysis of genomic sequences between the *rhcga* and *kif7* genes from Fugu, medaka and zebrafish was performed. Four highly conserved noncoding elements including CNE1, CNE2, CNE3 and CNE4 (sequence lengths >100 bp with > 75% identity) in a 90-Kb chromosomal region (Zv9, chromosome7: 15189000–15279000) of zebrafish genome were identified by the VISTA browser (Fig 6A). To test the function of these CNEs, we separately subcloned them to the right of the mMTI minimal promoter (Fig 6B) and examined their enhancer activities tested by injecting the plasmids into zebrafish embryos at one-cell stage. We found that 9% of embryos (58/650) injected with plasmids carrying the CNE2 exhibited specific EGFP expression in the midbrain, hindbrain and spinal cord at 48 hpf (Fig 6D and 6D’, EGFP) (Fig 6E and 6E’, WISH); expression was lost at later stage, which mimicked the EGFP expression patterns in embryos from ET line pTME12 at 48 hpf (Fig 6C and 6C’). However, EGFP expression driven by the mMTI basal promoter was not observed in the rest (91%) of embryos from 48 hpf to later stage. Embryos injected with plasmids containing CNE1, CNE3 or CNE4 revealed a ubiquitous expression, perhaps caused by transcriptional activity of the minimal promoter in the pTME vector. In addition, CNE2 is 1040bp away from the R-ITR of *miniTol2* transposon, (Fig 6A). These results demonstrate that CNE2 is a potential enhancer element that controls the activity of mMTI basal promoter for specific EGFP expression in the central nervous system of pTME12 transgenic fish.

### The mutation cassette of pTME vector functions in a transgenic line

**Fig 7:**
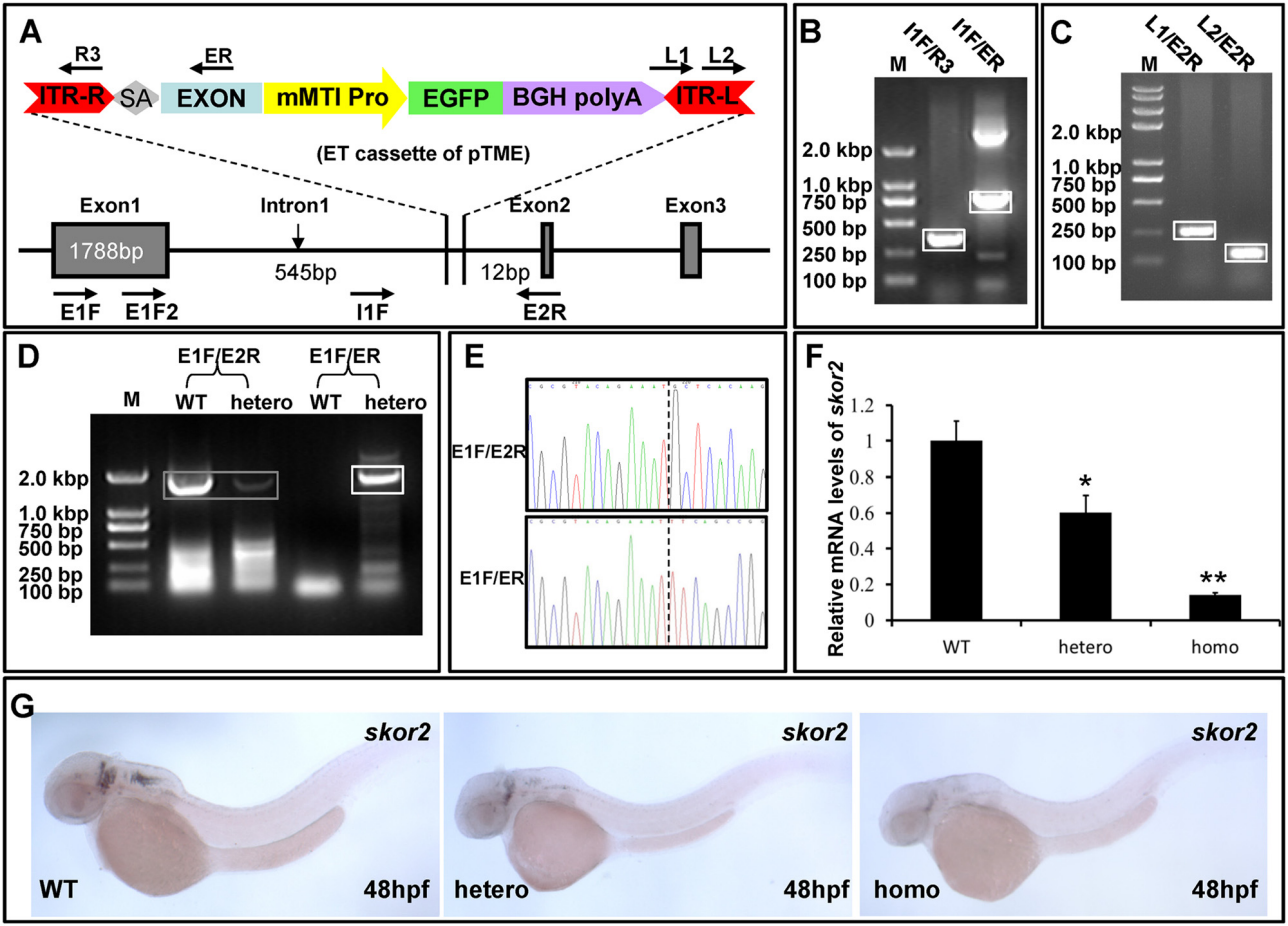
Insertion mutagenesis of gene *skor2* in a transgenic fish line. **(A**) A schematic representation of ET cassette inserted into the intron 1 of *skor2* gene. **(B and C)** Integrity analysis of ET cassette at the integration site by PCR using primers as indicated in (A). Boxed DNA bands (B and C) were separated and sequenced. **(D)** RT-PCR analysis of endogenous *skor2* and fusion transcripts in WT and heterozygous (hetero). Primer pairs E1F/E2R were used for amplification of endogenous *skor2* transcripts and E1F/ER for exon1-exon fusion transcripts. Two 1840-bp PCR products (boxed) were amplified with primer pairs E1F/E2R and represent a fragment of endogenous transcripts. A single 1856-bp PCR product (boxed) was amplified from heterozygous cDNA. **(E)** Sequencing data indicate the E1F/ER PCR product in (D) is a fusion transcript of *skor2* exon1 and a partial exon in pTME vector. Dotted line indicates the fusion position. **(F)** qRT-PCR was performed with primer E1F2/E2R to determine the expression of endogenous *skor2* gene in WT, heterozygous (hetero) and homozygous (homo) embryos at 48 hpf. The *skor2* expression levels were normalized to the *β-actin* levels. **(G)** WISH with *skor2* probes to detect the endogenous *skor2* mRNA expression in WT, heterozygous and homozygous embryos at 48 hpf.

ET cassettes were often found to land in an intron of some endogenous genes and the transcription of these genes is hardly affected by traditional ET trapping inserts [6, 7, 10, 12, 16]. These ET fish lines did not reveal any interesting expression patterns were usually discarded. To develop an ET vector that can disrupt with endogenous transcripts, we modified our ET vector, in which a splice acceptor (SA) was introduced to interrupt the normal splicing of endogenous genes once the ET cassette integrates into an intron. We found that the trapping cassette of pTME landed in the intron1 of gene *skor2* (ENSDARG00000063614) in an ET line with a background level of EGFP expression (Fig 7A and S1 Fig). PCR assays confirmed an intact ET cassette integrating in a region of 545 bp downstream of exon1 and 12 bp upstream of exon2 (Fig 7B and 7C).

To examine whether the mutation cassette functioned properly within intron1 of *skor2*, RT-PCR assays were conducted with cDNA samples from wide-type (WT, +/+) and heterozygous (+/-) embryos. Transcripts of endogenous *skor2* gene were detected in both WT and heterozygous embryos, while fusion transcripts of exon1 from *skor2* and an exon from mutation cassette appeared in heterozygous but not in WT embryos (Fig 7D). DNA sequencing indicated that the fusion transcript was derived from *skor2* exon1 and the exon in the mutation cassette (Fig 7E). Next, quantitative RT-PCR (qPCR) analysis was performed with cDNA samples from WT, heterozygous and homozygous F2 embryos at 48 hpf. As shown in Fig 7F, the *skor2* transcriptional levels in heterozygous and homozygous were significantly decreased in comparison with that in WT (an average decrease to 61% in heterozygous and 11% in homozygous). Additionally, WISH with *skor2* probes indicated that *skor2* expression in the hindbrain markedly reduced in heterozygous and homozygous embryos at 48 hpf (Fig 7G). These data indicate that the mutation cassette within the pTME vector is effective for disruption of gene transcription if the ET cassette has inserted into an intron of endogenous genes in a forward direction.

## Discussion

In this study, we have generated a novel ET vector that can be used for both ET screening and insertional mutagenesis in zebrafish. In the first round of screening, we obtained 12 stable ET fish lines with tissue or organ-specific EGFP expression from 235 individuals of founder fish (F0). The ET ratio (5%, = 12/235) appeared to be not as high as those in previous ET studies using *Tol2* transposon and other minimal promoters in zebrafish, i.e. 12% using the *keratin8* promoter and 160% using the *hsp70* promoter [12, 16]; however, 75 out of 235 F0 fish exhibited EGFP gene expression in their offspring and the transgenic rate (32%, = 75/235) was comparable to those in previous studies by Parinov *et al*. (2004) (16%) and Nagayoshi *et al*. (2008) (70%).

A biased interaction of minimal promoter with chromosomal enhancers was widely noticed during ET investigation since different promoters have distinct sensitivity in responsiveness to enhancer elements [46]. Fish lines from an ET vector carrying *keratin8* minimal promoter exhibited CNS-specific EGFP expression patterns, since the *krt8* promoter can drive the expression of *keratin8* gene in tissues originated from ectoderm and is thus effective to detect CNS-specific enhancers [16]. In another study, four distinct promoters for *hsp1*.*5*, *hsp0*.*6*, *E1b* and *c-fos* were separately used for construction of a *Tol2*-based ET vector. The *E1b* promoter displayed a very strong bias for cranial ganglia expression and most patterns obtained with this promoter looked similar [15]. In order to yield ET lines with biased specificity to neurons, a recent study generated an ET vector containing a biased minimal promoter *thy1mp*, which was isolated from the murine *Thy-1* gene and preferentially expressed in neurons of murine brain [47]. In the present study, we have obtained an ET line with specific EGFP expression in the pancreas (pTME10) and two lines with specific EGFP expression in the pronephric duct (pTME8 and pTME11). Organ-specific expression in a proportion of 25% (3/12) was rarely obtained from previous ET screening in zebrafish [6, 7, 48, 49]. The mMTI minimal promoter used was isolated from the murine *MT-I* gene, which is particularly expressed in liver, pancreas, intestine and kidney except for a ubiquitous distribution [50], and its minimal promoter may contain elements that restrict its activity in these parenchymatous organs. Therefore, most of minimal promoters currently used have a preference to detect enhancers that regulate gene expression in specific tissues or cells and seem to be insensitive to other chromosomal enhancers. Since the preference of ET vectors for specific tissues and cells can limit the efficiency of generating ET lines for saturation of ET screening across a target genome, alterative ET vectors including our pTME vector are needed to obtain as many enhancers as possible.

Although the distributions of *egfp* mRNA in pTME1, pTME3 and pTME9 largely mimicked the expression patterns of three endogenous genes, the transcription levels of *egfp* in the three lines were lower than that of corresponded endogenous genes in WT. There are two possibilities: 1) Embryos used for detection of *egfp* expression are derived from the heterozygotes of three lines and transcriptional levels of *egfp* in heterozygotes probably represent the expression from one copy of endogenous genes in WT embryos; 2) The transcription of a gene is controlled by the interaction of enhancers with the basal promoter. The expression of EGFP and endogenous gene were controlled by distinct basal promoter, so the EGFP expression in the ET lines depended on the interaction of mMTI basal promoter with potential enhancers near the insertion site, while the transcription of endogenous gene is controlled by enhancers and its own basal promoter. In addition, endogenous enhancers have distance- and orientation-independent characteristics [51] that allow the interaction of endogenous enhancers with the mMTI basal promoter, so the *egfp* expression demonstrated a pattern similar to that of corresponding endogenous genes.

A large number of highly conserved non-coding sequences between different species have been found through comparative genomics and half of these sequences are tissue-specific enhancers [41, 42, 52, 53]. In this study, we have identified conserved CNEs near an integration site in ET lines with specific EGFP expression. A CNE sequence adjacent to the insertion locus in line pTME12 exhibited activity in the brain and spinal cord, which largely recaptured the EGFP expression pattern in pTME12. We have also predicted three other nearest CNEs around the insertion sites in lines pTME1, pTME8 and pTME9; however, no visible EGFP expression was observed in zebrafish embryos injected with vectors containing these CNEs (data not shown). One possible explanation is that all of the three CNEs are silencers that negatively regulate *egfp* gene expression. Another reason is that the transient EGFP expression in injected embryos is not strong enough for vision, since EGFP expression in line pTME12 is extremely robust but only 9% of zebrafish embryos injected with a test vector containing CNE2 showed a transient and weak EGFP expression in the brain and spinal cord at 48 hpf. So, it’s necessary to further test the activity of other CNEs around integration locus in a transgenic zebrafish enhancer assay [26, 45, 54].

A previous study has shown that insertional mutagenesis can be created by traditional ET screening in zebrafish [12]; however, this ET-based mutation can only occur when the ET cassette is inserted into exons of endogenous genes. In this study, a mutation cassette containing a splice acceptor (SA) signal and a partial exon were introduced into the ET cassette of our ET vector, which can disrupt the transcription of targeted genes when the ET cassette integrates into an intron of endogenous gene in a forward direction. The SA and exon elements used in pTME vector were proved to work well in our previous research [25, 27]. Indeed, we found that the mutation cassette in our ET cassette worked efficiently to interrupt the expression of *skor2* gene in a transgenic zebrafish line. However, in a previous study, the combination of SA and exon was unable to disrupt the endogenous gene expression and a strong transcriptional terminator should be fused to the mutagenicity cassette [55]. We presume that the modest mutagenicity of pTME elements is due to the effect of SA and partial exon as well as the role of BGH poly(A) signal in the ET cassette. Our RT-PCR results demonstrated that the fusion transcripts (splice variant I in S2 Fig) in *skor2* ET/GT homozygotes contained the exon1 from *skor2*, an exon from mutation cassette and the whole ET cassette (Fig 7D and S2 Fig). These data indicate that the BGH poly(A) signal in the ET cassette functions as a terminator in the transcription of the fusion gene, and thus can improve the efficiency of interrupting the expression of endogenous genes. Another SA and a strong transcriptional terminator will be introduced into the right end of the ET cassette to mutate a gene when the ET cassette integrates into the genome in a reverse direction.

In summary, we have generated a novel ET vector that can be used for both enhancer trapping and insertion mutagenesis across a target genome.

## Supporting Information

S1 Checklist ARRIVE guidelines checklist. (DOCX)

S1 Fig EGFP expression patterns in the fish line with a disrupted expression of skor2 gene. **(A and B)** Lateral (A) and dorsal (B) view of embryos at 48hpf.
(TIF)

S2 Fig Transcriptional analysis of the trapping cassette in the intron 1 of gene *skor2*. **(A and B)** A schematic representation of potential splice variants in *skor2* ET/GT homozygotes. Insertion of pTME cassette into the first intron of gene *skor2* is illustrated in A. Splice variant I represents a fusion transcript of the endogenous exon 1 and the pTME cassette-derived sequence and this fusion transcript is stabilized by the BGH poly (A) signal in the pTME cassette. Splice Variant II represents the transcription of endogenous gene *skor2*. **(C)** RT-PCR analysis of transcripts from *skor2* ET/GT homozygotes. Primers E1F2/EGR and E1F2/BR were used to amplify the fusion transcript. Sequencing results indicate the 1164 bp and 467 bp bands are derived from the splice variant I.
(TIF)
