# Supplementary material for: Generation of an Enhancer-Trapping Vector for Insertional Mutagenesis in Zebrafish
Source: PLoS One. 2015 Oct 5;10(10):e0139612. doi: 10.1371/journal.pone.0139612 (PMC4593583; doi:10.1371/journal.pone.0139612)

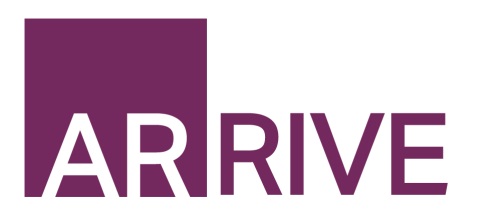


The ARRIVE Guidelines Checklist

Animal Research: Reporting In Vivo Experiments

Carol Kilkenny^1^, William J Browne^2^, Innes C Cuthill^3^, Michael Emerson^4^ and Douglas G Altman^5^

*^1^The National Centre for the Replacement, Refinement and Reduction of Animals in Research, London, UK, ^2^School of Veterinary Science, University of Bristol, Bristol, UK, ^3^School of Biological Sciences, University of Bristol, Bristol, UK, ^4^National Heart and Lung Institute, Imperial College London, UK, ^5^Centre for Statistics in Medicine, University of Oxford, Oxford, UK.*

|  | | ITEM | RECOMMENDATION | Section/ Paragraph |
| --- | --- | --- | --- | --- |
| 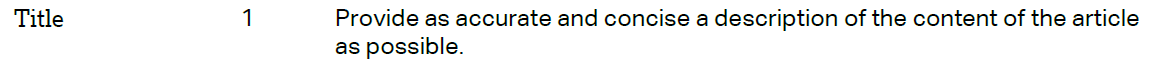 | | | Title |  |
| 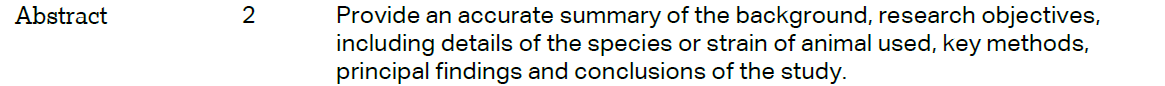 | | | Abstract |  |
| INTRODUCTION | | |  |  |
| 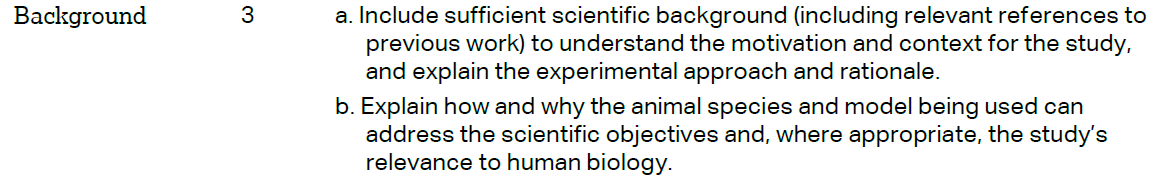 | | | Paragraphs 1-4  Paragraph 2 |  |
| 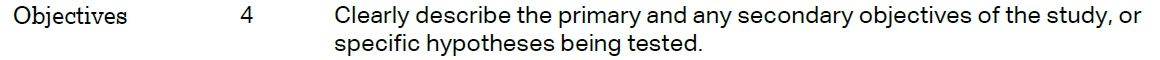 | | | Paragraphs 3-5 |  |
| METHODS | | |  |  |
| 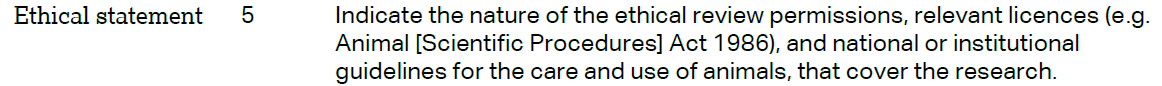 | | | Under Ethical Statement |  |
| 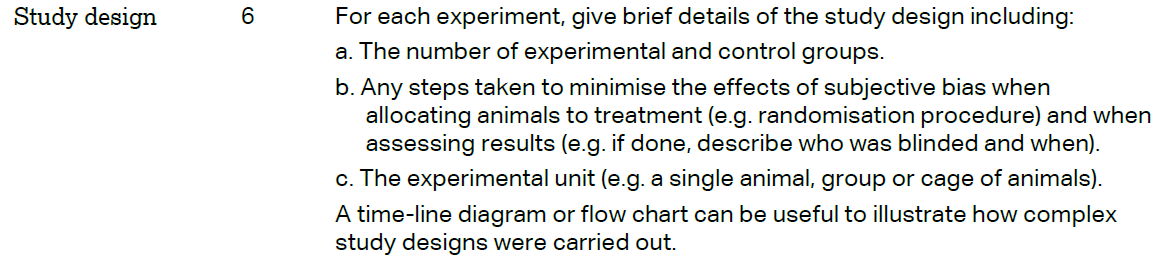 | | | In‘Microinjecti-on and selection of EGFP-expressing F1 fish’  Paragraph 1 |  |
| 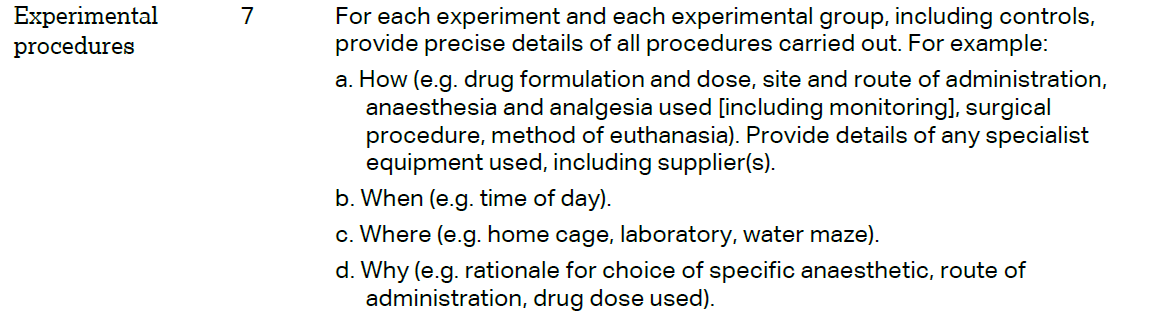 | | | In ‘Microinjection and selection of EGFP-expressing F1 fish’and  ‘Genome walking and PCR assays’ |  |
| 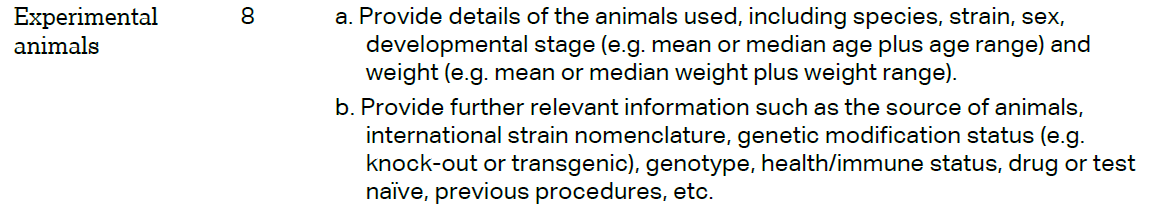 | | | In ‘Zebrafish husbandry’ |  |

The ARRIVE guidelines. Originally published in *PLoS Biology*, June 2010^1^

| 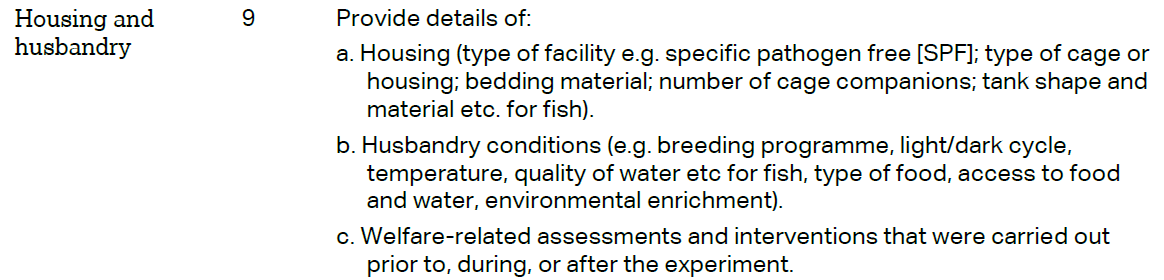 | In ‘Zebrafish husbandry’ | |
| --- | --- | --- |
| 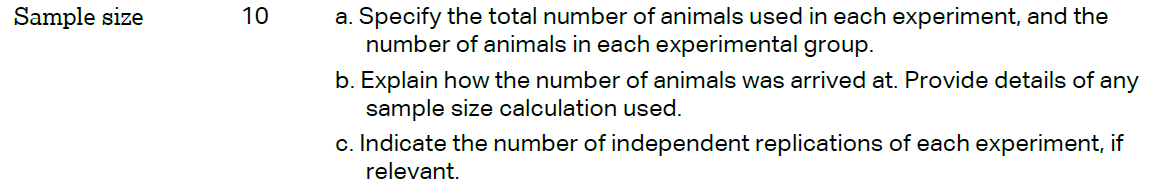 | In‘Microinjecti-on and selection of EGFP-expressing F1 fish’  and Table1 | |
| 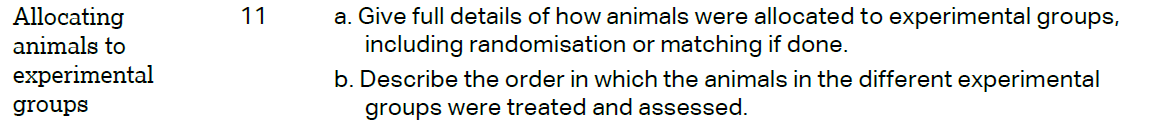 | In‘Microinjecti-on and selection of EGFP-expressing F1 fish’ | |
| 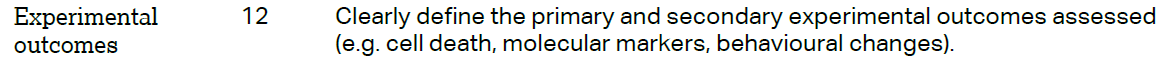 | n/a | |
| 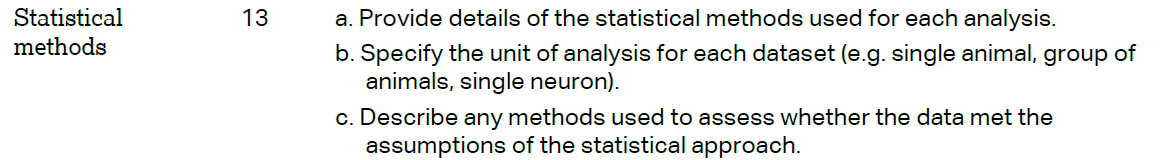 | In‘Genome walking and PCR assays’ Paragraph 3 and  ‘Statistical Analysis’ | |
| RESULTS |  | |
| 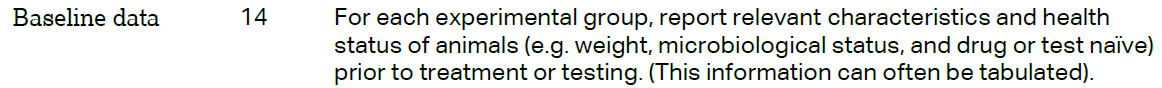 | n/a | |
| 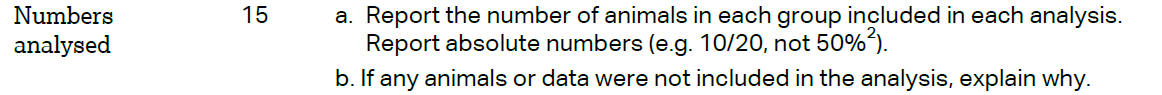 | In Results  Paragraph3,17 and Table1 | |
| 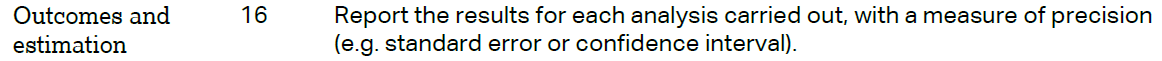 | In results and Figure 1,2,7 | |
| 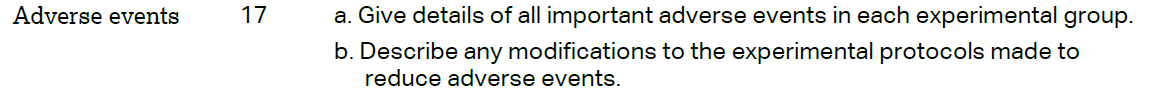 | n/a | |
| DISCUSSION |  | |
| 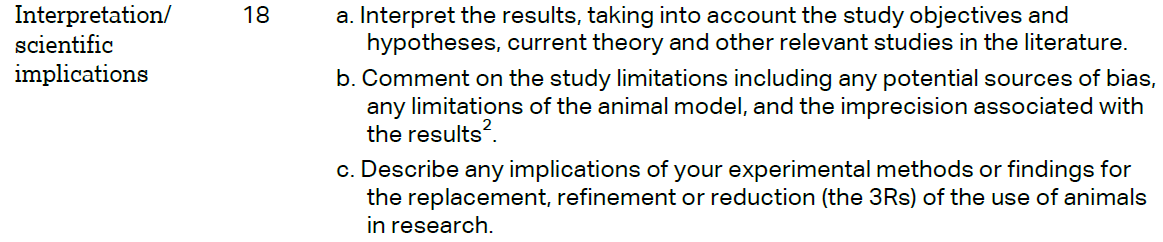 | Discussion | |
| 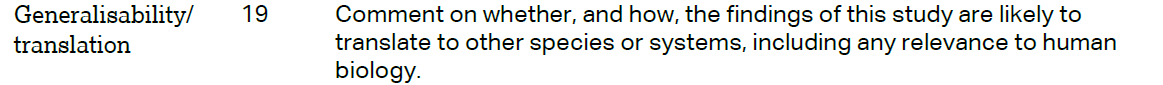 | n/a | |
| 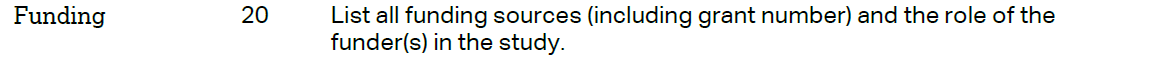 | | Funding Statement |


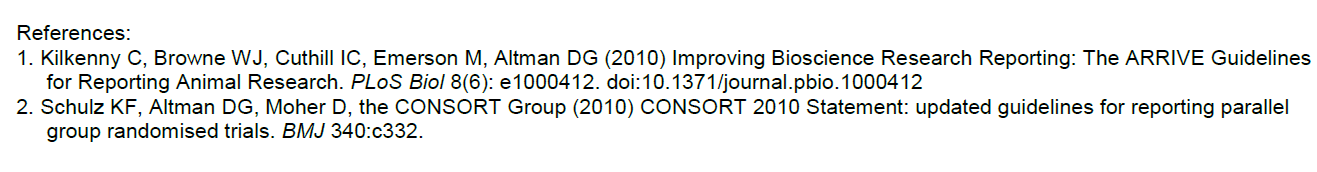

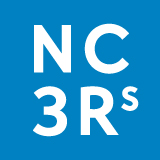

Supplement: S1 Checklist — (DOCX) [file pone.0139612.s001.docx]
