# Supplementary figures and images for: Generation of an Enhancer-Trapping Vector for Insertional Mutagenesis in Zebrafish
Source: PLoS One. 2015 Oct 5;10(10):e0139612. doi: 10.1371/journal.pone.0139612 (PMC4593583; doi:10.1371/journal.pone.0139612)

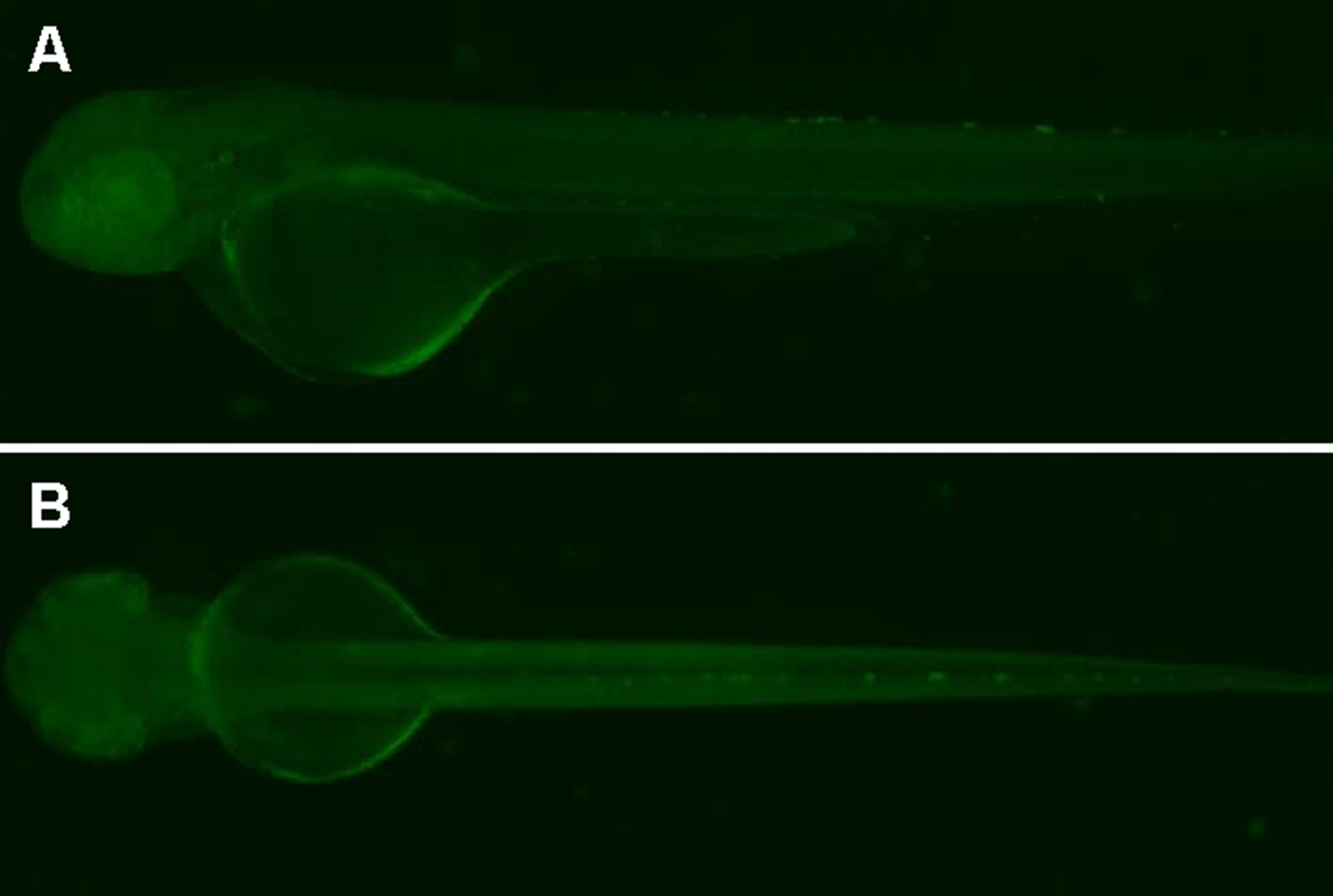

Supplement: S1 Fig — (A and B) Lateral (A) and dorsal (B) view of embryos at 48hpf. (TIF) [file pone.0139612.s002.tif]

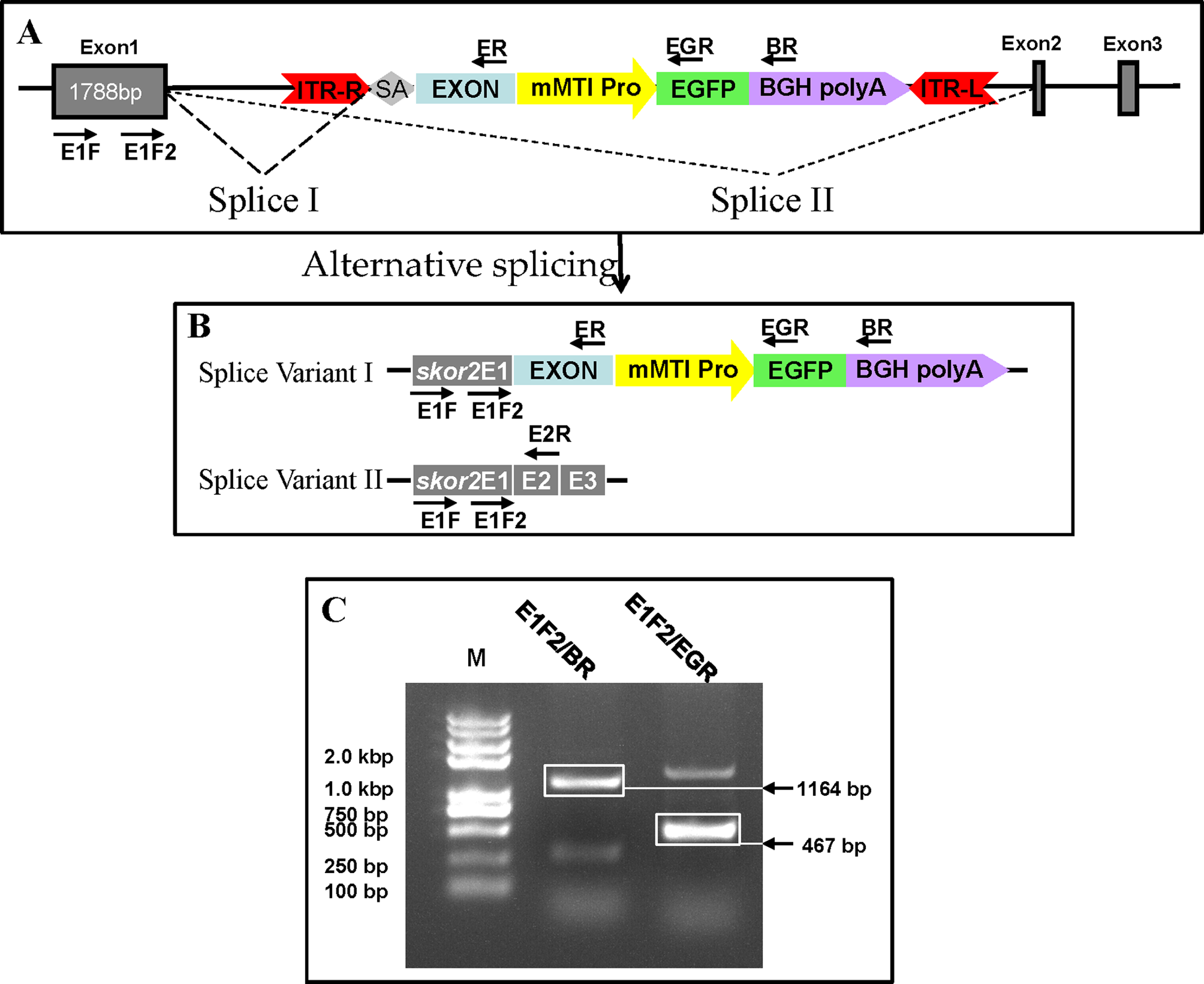

Supplement: S2 Fig — (A and B) A schematic representation of potential splice variants in skor2 ET/GT homozygotes. Insertion of pTME cassette into the first intron of gene skor2 is illustrated in A. Splice variant I represents a fusion transcript of the endogenous exon 1 and the pTME cassette-derived sequence and this fusion transcript is stabilized by the BGH poly (A) signal in the pTME cassette. Splice Variant II represents the transcription of endogenous gene skor2. (C) RT-PCR analysis of transcripts from skor2 ET/GT homozygotes. Primers E1F2/EGR and E1F2/BR were used to amplify the fusion transcript. Sequencing results indicate the 1164 bp and 467 bp bands are derived from the splice variant I. (TIF) [file pone.0139612.s003.tif]
